# Ursolic acid inhibits epithelial-mesenchymal transition *in vitro* and *in vivo*

**DOI:** 10.1080/13880209.2019.1577464

**Published:** 2019-03-24

**Authors:** Chang-Geng Xu, Xia-Lian Zhu, Wei Wang, Xiang-Jun Zhou

**Affiliations:** a Department of Urology, The Central Hospital of Wuhan, Tongji Medical College, Huazhong University of Science and Technology, Wuhan, China;; b Department of Hand Surgery, Affiliated Nanhua Hospital of University of South China, Hengyang, China;; c Department of Urology, The First Affiliated Hospital of Anhui Medical University, Hefei, China;; d Department of Urology, Renmin Hospital of Wuhan University, Wuhan, China

**Keywords:** Renal tubulointerstitial fibrosis, transforming growth factor beta1, cell dedifferentiation, animals

## Abstract

**Context**: Ursolic acid (UA; 3β-hydroxy-urs-12-en-28-oic acid), one of the pentacyclic triterpenoids found in various plants and herbs, possesses some beneficial effects under pathological conditions, including combating hepatic fibrosis.

**Objective**: This study investigates the effects of UA on renal tubulointerstitial fibrosis *in vivo* and *in vitro*.

**Materials and methods**: *In vivo*, 24 male C57BL6 mice were divided into four groups. Eighteen mice were subjected to unilateral ureteral obstruction (UUO) and the remaining six sham-operated mice served as control. UUO mice received either vehicle or UA (50 or 100 mg/kg) by gastric gavage for 6 days. *In vitro*, HK-2 cells were treated with 10 or 50 μM UA and 10 ng/mL recombinant human transforming growth factor-β1 (TGF-β1). The molecular mechanisms of fibrosis were investigated.

**Results**: UUO induced marked interstitial collagen I and fibronectin deposition and epithelial-mesenchymal transition (EMT), as evidenced by increased α-smooth muscle actin (α-SMA) and decreased E-cadherin. However, UA treatment significantly reduced collagen I and fibronectin accumulation in the fibrotic kidney. UA treatment also decreased α-SMA and preserved E-cadherin *in vivo*. *In vitro*, TGF-β1-treated HK-2 cells demonstrated elevated α-SMA, snail1, slug, TGF-β1, and p-smad3, as well as diminished E-cadherin. UA pretreatment prevented E-cadherin loss and diminished α-SMA expression in HK-2 cells. UA downregulated mRNA expression of snail1 and slug. UA also lowered TGF-β1 protein expression and p-Smad3 in HK-2 cells.

**Conclusions**: UA attenuated renal tubulointerstitial fibrosis by inhibiting EMT, and such inhibition may be achieved by decreasing profibrotic factors. UA may be a novel therapeutic agent for renal fibrosis.

## Introduction

Renal fibrosis is an inevitable outcome of nearly all kinds of chronic kidney diseases (CKD). Despite the removal of initial insults, renal lesions are not relieved, but progress toward end-stage renal disease (ESRD) (Zhou et al. 2013). The mechanism that promotes the progression of renal injury remains unclear. Epithelial-mesenchymal transition (EMT), which gives rise to the matrix-producing myofibroblasts, is one of the essential pathological processes in renal fibrogenesis (Carew et al. 2012). During kidney fibrosis in mice, approximately 36% myofibroblasts derived from the tubular epithelial cells of the kidney through EMT (Iwano et al. 2002). Clinical studies utilizing human kidney biopsies also suggest that EMT likely plays vital roles in the pathogenesis of human CKD (Hertig et al. 2008). Therefore, inhibition of EMT may halt the progression of CKD.

Angiotensin-converting enzyme inhibitors and angiotensin receptor blockers are mainstream therapeutic strategies for CKD (Wolf and Ritz 2005). However, these medications have serious side effects. Furthermore, resistance to therapy and relapse of disease after discontinuation of medication are common. For patients with CKD, modulation of the renin-angiotensin axis provides only partial salutary effects and does not necessarily prevent the progression to ESRD (Seva Pessoa et al. 2013). Natural medicines have shown their efficacy in the clinical field, although the underlying mechanisms remain undefined (Jiang et al. 2013). The lack of therapeutic options for CKD has prompted us to seek alternative treatments, such as natural medicines and clarify their possible mechanisms.

Ursolic acid (UA), a natural pentacyclic triterpenoid, is widely distributed in food, medicinal herbs and other plants. It possesses many biological activities, including antitumor, antioxidant, and anti-inflammatory properties (Sultana 2011). UA shows various beneficial effects under pathogenic conditions and exhibits low toxicity (Jin et al. 2012; Pai et al. 2012). UA ameliorated hepatic fibrosis either by induction of apoptosis or inhibition of activation of hepatic stellate cells (Shyu et al. 2008; Wang et al. 2011). UA also inhibited the development of glomerular hypertrophy and type collagen IV accumulation in streptozotocin-induced diabetic mice (Zhou et al. 2010). Importantly, UA is an antagonist for transforming growth factor-β1 (TGF-β1) signalling (Murakami et al. 2004). Given the important roles of TGF-β1 in fibrogenesis and EMT, we hypothesized that UA can slow down the progression of tubulointerstitial fibrosis, mainly focusing on EMT.

## Materials and methods

### Animals and antibodies

Male C57BL6 mice (18–20 g) were purchased from the Experimental Animal Center of Wuhan University (Wuhan, China). Animals were housed at a constant temperature and with a consistent dark/light cycle. All surgical and experimental procedures were approved by the Institutional Animal Care and Use Committee of Wuhan University. UA was obtained from Shanghai Yuanye Biotechnology (Shanghai, China). It was suspended in appropriate concentrations with carboxymethyl cellulose before use in experiments. Anti-collagen I (ab34710), fibronectin (ab2413), α-SMA (ab5694), TGF-β1 (ab92486) antibodies were purchased from Abcam (Cambridge, UK); Anti-p-Smad3 (pS423/425) antibody was purchased from Epitomics (Cambridge, UK); Anti-E-cadherin (24E10) antibody was purchased from cell signalling technology (Boston, MA, USA); Anti-actin (sc-7210) antibody was purchased from Santa Cruz (Dallas, TX, USA). Secondary antibody conjugated with IRDye^®^ infrared dyes was purchased from Rockland (Rockland Immunochemicals, Inc).

### Experimental protocol

Twenty-four male C57BL6 mice were randomly divided into four groups, with six mice per group: (a) Sham group (Sh); (b) unilateral ureteral obstruction (UUO) plus vehicle group (UUO + V); (c) UOO plus 50 mg/kg body weight UA (UUO + D1); (d) UOO plus 100 mg/kg body weight UA (UUO + D2). UUO was performed as described previously (Chevalier et al. 2009). In brief, under sodium pentobarbital (60 mg/kg body weight) anaesthesia, complete ureteral obstruction was performed by ligating the left ureter with 4–0 sutures, following a left abdominal incision. Sham-operated mice had their ureter exposed and manipulated, but not ligated. UA of 50 or 100 mg/kg body weight (suspended in 0.5% carboxymethyl cellulose) or an equal volume of vehicle (phosphate buffered saline dissolved in 0.5% carboxymethyl cellulose) was orally administered from day 1 to day 6 following operations. Mice were sacrificed on the 7th day, and the obstructed kidneys were harvested. Half of each kidney was fixed in 4% buffered paraformaldehyde and embedded in paraffin for histological and immunohistochemical studies. The remaining kidneys were snap-frozen in liquid nitrogen and stored at −80 °C for protein and RNA extraction.

### Histological and immunohistochemical examination

Kidney sections from paraffin-embedded tissues were prepared at 5 μm thickness. Total collagen was identified by picric sirius red (PSR) staining through a method from a previous report with some modification (Jiang et al. 2013). For immunohistochemical examination, the renal sections were incubated with anti-collagen I antibody (1:100), or anti-fibronectin antibody (1:100), or anti-α-SMA antibody (1:200), or anti-E-cadherin antibody (1:400). The incubation of primary antibodies was carried out overnight at 4 °C, followed by a second reaction with anti-rabbit antibody conjugated with envision polymer for 30 min. Finally, a diaminobenzidine reaction was performed on the section using a kit (Thermo Scientific, TL-015-QHD) and hematoxylin was used as the counterstain.

### Western blot analysis

Western blot analysis was performed as described in a previous study (Wu et al. 2013). In brief, protein samples were heated at 100 °C for 5 min and subjected to electrophoresis on 10% sodium dodecyl sulphate-polyacrylamide gels. Proteins were electrophoretically transferred to nitrocellulose membranes which were incubated with antibodies specific for a-SMA (1:500), E-cadherin (1:1000), TGF-β1 (1:400), p-Smad3 (1:1000) and actin (1:1000), followed by incubated secondary antibody conjugated with IRDye^®^ infrared dyes. The signals were detected with Odyssey (Li-Cor Biosciences, Lincoln, NE, USA). Actin was used as an internal control.

### Real-time PCR

Quantitative real-time polymerase chain reaction (qRT-PCR) following reverse transcription was used to assess the transcript levels of collagen I, fibronectin, snail1 and slug. RNA was extracted from the frozen tissue by homogenization in Trizol (Invitrogen Life Technologies), and 1 μg aliquots of RNA was used in reverse transcription reaction with M-MuLV reverse transcriptase (Thermo Scientific, #K1621). The resulting cDNA was used as template for PCR analysis. Primers were obtained from Sangon Biological Engineering Technology and Services (Shanghai, China), specific primers were designed as follows: (1) TGCCGCGACCTCAAGATGTG (sense) and CACAAGGGTGC TGTAGGTGA (antisense) for collagen I; (2) CTTCTCCGTGGAG TTTTACCG (sense) and GCTGTCAAATTGAATGGTGGTG (antisense) for fibronectin; (3) GAGGACAGTGGCAAAAGCTC (sense) and CGGATGTGCATCTTCAGAG (antisense) for snail1; (4) AACACACACTGGGGAAAAGC (sense) and ACAGCAGCCA GACTCCTCAT (antisense) for slug; and (5) GGTGAAGGTCG GTGTGAACG (sense) and CTCGCTCCTGGAAGATGGTG (antisense) for GAPDH. A 20 μL sample of real-time PCR reaction solution, which included SYBR Green PCR Master Mix (TaKaRa, #RR420A) was amplified according to the manufacturer’s instructions. Real-time quantifications were performed in duplicate on the ABI PRISMW 7500 Sequence Detection System (Applied Biosystems). The calculation of relative change in mRNA was performed using the delta-delta method (Livak and Schmittgen 2001).

### Cell culture and treatment

Human proximal tubular epithelial cell (HK-2 cell) was purchased from the Cell Resource Center of the Shanghai Institutes for Biological Sciences Chinese Academy of Sciences (Shanghai, China), which was originally obtained from ATCC. To evaluate the inhibitory effect of UA on the phenotypic transformation of tubular epithelial cells into myo-fibroblastic appearance induced by TGF-β1, HK-2 cells were seeded at 2 × 10^5^/mL in six-well plates in DMEM-F12 medium supplemented with 10% fetal bovine serum (FBS) for 24 h. Then cells were cultured in FBS-free medium for another 24 h. Cells were pretreated with 10 or 50 μM UA for 4 h and then incubated with 10 ng/mL recombinant human TGF-β1 (R&D Systems) for 4 or 48 h. The cells were photographed under bright light or harvested for protein and RNA extraction.

### Statistical analysis

All data were expressed as the mean ± standard deviation. Statistical analysis was performed using the SPSS 18.0 software (SPSS Inc., Chicago, USA). Intergroup comparisons were made by one-way analysis of variance, followed with Bonferroni’s test. The results were considered to be significant at *p* < 0.05.

## Results

### UA attenuates UUO-induced tubulointerstitial fibrosis

**Figure 1.**
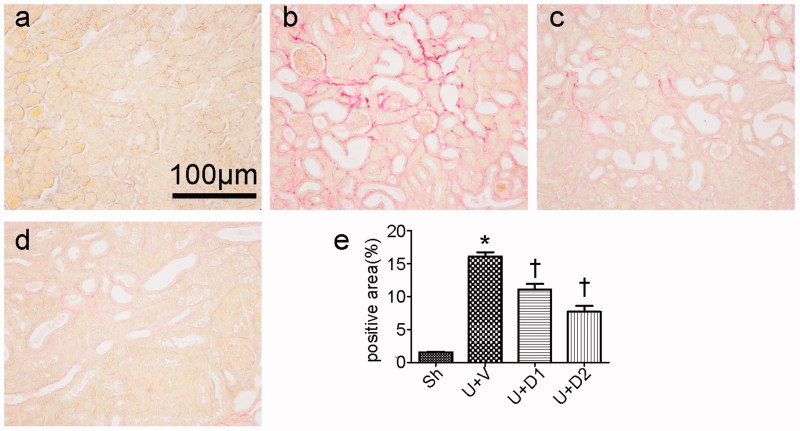
Representative photographs of PSR staining. (a) Sham group (Sh); (b) UUO plus vehicle group (UUO + V); (c) UOO plus 50 mg/kg UA (UUO + D1); (d) UOO plus 100 mg/kg UA (UUO + D2). (e) Semiquantitative analysis of positive area. *p < 0.05 VS. Sh, †*p* < 0.05 VS. U + V. Scale bar = 100 μm.

**Figure 2.**
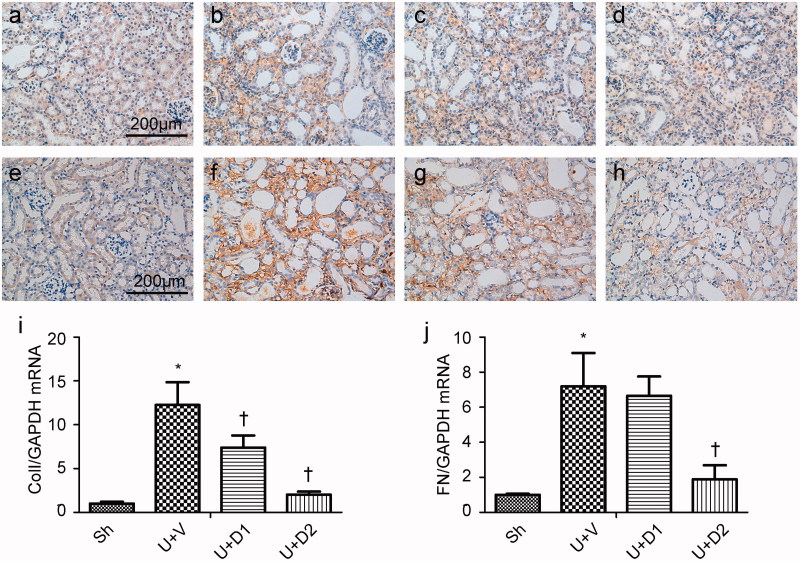
Representative photographs of collagen I (a–d) and fibronectin (e–h) staining. (a, e) Sh; (b, f) U + V; (c, g) U + D1; (d, h) U + D2. (i, j) Semiquantitative PCR analysis for mRNA expression of collagen I and fibronectin. *p < 0.05 VS. Sh, †*p* < 0.05 VS. U + V. Scale bar = 200 μm.

Total collagen deposition was evaluated by PSR staining 7 days after ureteral obstruction. As shown in Figure 1, compared with sham operations, UUO induced massive accumulation of collagen in tubulointerstitium (16.06 ± 0.68% vs. 1.56 ± 0.09%, *p* < 0.05). However, the degree of fibrosis was alleviated in the UUO + D1 (11.09 ± 0.83% vs. 16.06 ± 0.68%, *p* < 0.05) and UUO + D2 (7.69 ± 0.90% vs. 16.06 ± 0.68, *p* < 0.05) groups. The increased extracellular matrix is mainly composed of collagen and fibronectin, so, we examined the expression of collagen I and fibronectin. As presented in Figure 2, a significantly greater amount of interstitial matrix was observed in the UUO + V group compared with sham group. UA treatment decreased the deposition of collagen I and fibronectin by immunohistochemical staining. Furthermore, the inhibitory effect of UA on fibrosis was verified by qRT-PCR. UUO induced increased expression of collagen I (12.26 ± 2.59 vs. 1 ± 0.19), treatment with low and high dose of UA diminished collagen I expression (7.39 ± 1.39 vs. 12.26 ± 2.59, 2.03 ± 0.35 vs. 12.26 ± 2.59, *p* < 0.05, respectively). UUO also leaded to the enhanced expression of fibronectin (7.20 ± 1.9 vs. 1 ± 0.06, *p* < 0.05) and high dose of UA resulted in a considerable reduction in its expression (1.89 ± 0.8 vs. 7.20 ± 1.9, *p* < 0.05).

### UA decreases UUO-induced epithelial-mesenchymal transition

**Figure 3.**
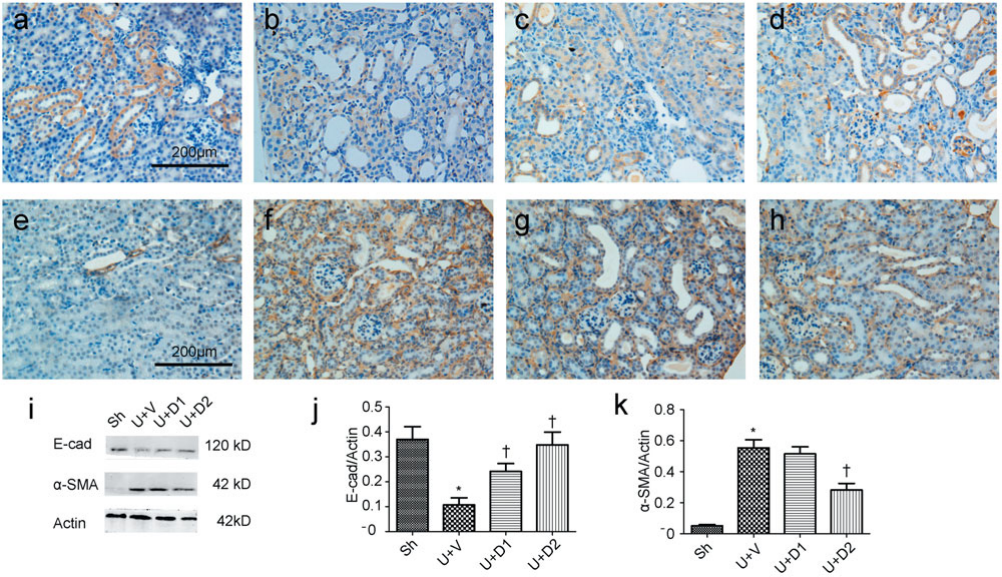
Representative photographs of E-cadherin (a-d) and α-SMA (e-h) staining. (a, e) Sh; (b, f) U + V; (c, g) U + D1; (d, h) U + D2. (i) Western blot analysis for E-cadherin and α-SMA. (j, k) Semiquantitative analysis for protein expression of E-cadherin and α-SMA. *p < 0.05 VS. Sh, †*p* < 0.05 VS. U + V. Scale bar = 200 μm.

EMT is an essential pathological process in tubulointerstitial fibrosis, which involves the loss of cell-cell membrane contact and the gain of mesenchymal biomarker. The transformed epithelium can produce a huge amount of extracellular matrix. Thus, we examined if UA can decrease UUO-induced EMT. As shown in Figure 3, in normal kidney, high amounts of E-cadherin locate at the plasma membrane and low amounts of α-SMA surround the vessels. UUO prevented the expression of E-cadherin and increased the expression of α-SMA; however, treatment with UA corrected these changes. Western blot analysis for E-cadherin and α-SMA corroborated these findings. Compared with sham group, UUO induced decreased expression of E-cadherin (0.37 ± 0.05 vs. 0.11 ± 0.03, *p* < 0.05), whereas low and high dose of UA rescued it (0.24 ± 0.03 vs. 0.37 ± 0.05, 0.35 ± 0.05 vs. 0.37 ± 0.05, respectively, *p* < 0.05). UUO also induced increased expression of α-SMA (0.55 ± 0.05 vs. 0.05 ± 0.01, *p* < 0.05), whereas high dose of UA can significantly reduce the expression of α-SMA (0.28 ± 0.04 vs. 0.55 ± 0.05, *p* < 0.05), although low dose of UA failed to do so (0.52 ± 0.05 vs. 0.55 ± 0.05, *p* > 0.05).

### UA ameliorates epithelial-mesenchymal transition in vitro

**Figure 4.**
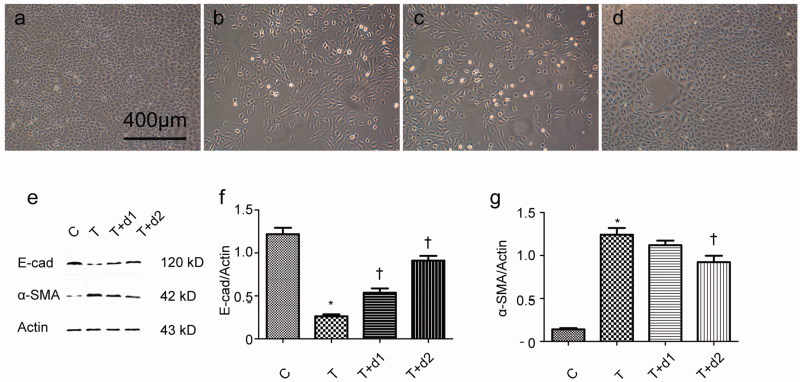
Representative photographs of cell morphology. (a) HK-2 treated with media only; (b) HK-2 treated with TGF-β1 (10 ng/mL); (c) HK-2 treated with TGF-β1 (10 ng/mL) and UA (10 μM); (d) HK-2 treated with TGF-β1 (10 ng/mL) and UA (50 μM). (e) Western blot analysis for E-cadherin and α-SMA. (f, g) Semiquantitative analysis for protein expression of E-cadherin and α-SMA. *p < 0.05 VS. C, †*p* < 0.05 VS. T. Scale bar = 400 μm.

**Figure 5.**
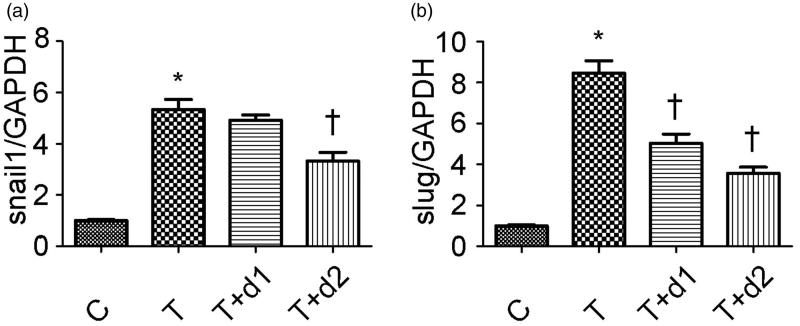
Semiquantitative analysis for mRNA expression levels of snail1 and slug. C: HK-2 treated with media only; T: HK-2 treated with TGF-β1 (10 ng/mL); T + d1: HK-2 treated with TGF-β1 (10 ng/mL) and UA (10 μM); T + d2: HK-2 treated with TGF-β1 (10 ng/mL) and UA (50 μM). *p < 0.05 VS. C, †*p* < 0.05 VS. T.

After treatment with TGF-β1, tubule epithelium transforms into mesenchymal cell. We examined if UA can ameliorate EMT *in vitro*. As shown in Figure 4, without TGF-β1 treatment, tubular cells demonstrated typical cobblestone pattern. However, the cells displayed a fibroblast-like morphology identifiable by the presence of elongated lamellipodia and a spindle shape after TGF-β1 treatment. Pretreatment with UA corrected the morphologic changes caused by TGF-β1. Consistent with these findings, Western blot analysis showed that the decreased E-cadherin expression (0.26 ± 0.06 vs. 1.22 ± 0.18, *p* < 0.05) after TGF-β1 exposure was restored by UA pretreatment (0.54 ± 0.12 vs. 0.26 ± 0.06, 0.91 ± 0.14 vs. 0.26 ± 0.06, respectively, *p* < 0.05). α-SMA expression was upregulated when the cells were exposed to TGF-β1 (1.24 ± 0.19 vs. 0.14 ± 0.04, *p* < 0.05). However, pretreatment with 50 μM UA significantly decreased the TGF-β1-induced upregulation of α-SMA (0.92 ± 0.18 vs. 1.24 ± 0.19, *p* < 0.05). Two key transcription factors, snail1 and slug, are essential for repressing E-cadherin and triggering EMT. Hence, we examined if UA can affect the expression of snail1 and slug. As shown in Figure 5, qRT-PCR revealed the increased snail1 expression (5.34 ± 0.95 vs. 1.00 ± 0.08, *p* < 0.05) after TGF-β1 exposure was decreased by 50 μM UA pretreatment (3.32 ± 0.84 vs. 5.34 ± 0.95, respectively, *p* < 0.05). Moreover, the increased slug expression (8.46 ± 1.47 vs. 1.00 ± 0.13, *p* < 0.05) after TGF-β1 exposure was also diminished by UA pretreatment (5.04 ± 1.10 vs. 8.46 ± 1.47, 3.58 ± 0.72 vs. 8.46 ± 1.47, respectively, *p* < 0.05).

### UA decreases smad-dependent TGF-β1 signalling

**Figure 6.**
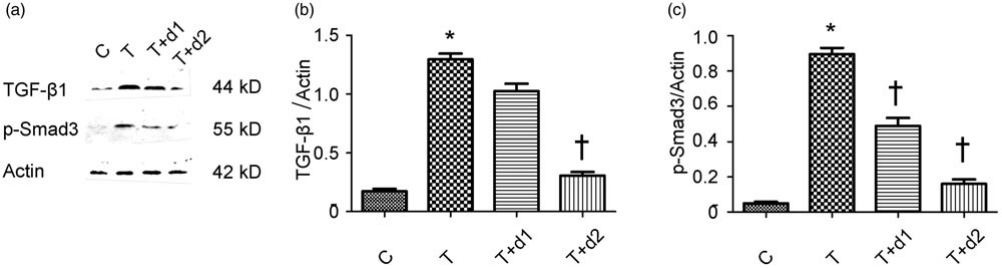
(a) Representative gel photographs of Western blotting for TGF-β1 and p-Smad3. (b, c) Semiquantitative analysis for protein expression of TGF-β1 and p-Smad3. C: HK-2 treated with media only; T: HK-2 treated with TGF-β1 (10 ng/mL); T + d1: HK-2 treated with TGF-β1 (10 ng/mL) and UA (10 μM); T + d2: HK-2 treated with TGF-β1 (10 ng/mL) and UA (50 μM). *p < 0.05 VS. C, †*p* < 0.05 VS. T.

Given the vital roles of Smad-dependent TGF-β1 signalling in the fibrogenesis, we examined if UA can downregulate the expression of TGF-β1 and phosphorylated Smad3. As shown in Figure 6, Western blot revealed the increased TGF-β1 expression (1.30 ± 0.12 vs. 0.18 ± 0.05, *p* < 0.05) after TGF-β1 exposure was decreased by 50 μM UA pretreatment (0.31 ± 0.07 vs. 1.30 ± 0.12, *p* < 0.05). In addition, the increased phosphorylated Smad3 (0.90 ± 0.09 vs. 0.05 ± 0.02) after TGF-β1 exposure was significantly diminished by UA pretreatment (0.49 ± 0.11 vs. 0.90 ± 0.09, 0.16 ± 0.06 vs. 0.90 ± 0.09, respectively, *p* < 0.05).

## Discussion

Renal fibrosis is a common final way for a wide variety of progressive CKD (Zhou et al. 2013). In normal kidneys, fibroblasts are quiescent and scarce in numbers and they are situated in the interstitial space between the capillaries and tubular epithelium. Upon activation by pro-fibrotic cytokines or mechanical stress *in vivo*, fibroblasts acquire a myofibroblast phenotype by expressing α-SMA and generate a large amount of extracellular matrix (ECM) components (Liu 2010). The size of the myofibroblasts is a major determinant of renal fibrotic lesions and kidney dysfunction (Kalluri and Neilson 2003). Myofibroblasts in injured kidneys derive from different sources through diverse mechanisms, including activation of interstitial fibroblasts and pericytes, phenotypic conversion of tubular epithelial and endothelial cells, as well as recruitment from circulating fibrocytes (Boor et al. 2010).

EMT is a biological process that allows a polarized epithelial cell, which normally interacts with basement membrane through its basal surface, to undergo multiple biochemical changes that enable it to assume a mesenchymal cell phenotype. EMTs are encountered in three distinct biological settings that carry different functional consequences (Thiery et al. 2009). Type 2 EMT is associated with wound healing and tissue regeneration. More specifically, such EMT is associated with fibrosis occurring in kidney, liver, lung, and intestine (Piera-Velazquez et al. 2011). Lineage-tracing experiments demonstrated that approximately 36% myofibroblasts derived through EMT from the injured tubular epithelial cells of the kidney (Iwano et al. 2002). Several studies have shown fibrosis amelioration by targeting EMT (Bani-Hani et al. 2009; Piera-Velazquez et al. 2011; Ding et al. 2012). Consistent with these studies, we found that UA can inhibit α-SMA upregulation and restore E-cadherin level *in vivo* fibrosis model, which was revealed by immunohistochemical staining and Western blot analysis. Moreover, in an *in vitro* model, after treatment with TGF-β1, HK-2 showed morphological changes, from cobblestone to spindle appearance. UA treatment rescued this morphological change. Western blot analysis also corroborated EMT, as evidenced by the phenomenon in which when HK-2 was incubated with TGF-β1 and UA, UA can prevent the gain of α-SMA and the loss of E-cadherin. These findings strongly indicated that UA can inhibit EMT.

Snail1, which belongs to the snail family of zinc-finger transcription factors, is a strong repressor of E-cadherin and implicated in EMT (Rowe et al. 2011). A previous study showed that snail1 was upregulated in the tubular epithelial cells of the obstructed kidneys in a rat model of UUO and in human proximal tubule HKC-8 cells treated with TGF-β1 and demonstrated that snail1 is involved in renal tubular EMT (Yoshino et al. 2007). Another study demonstrated that snail1 is closely related to EMT in human renal grafts (Xu-Dubois et al. 2013). Our data suggested that UA arrested the EMT program in UUO kidneys, thereby inhibiting interstitial fibrosis in obstructive nephropathy. Slug transcription factor, another repressor of E-cadherin promoter, has a well-established role in EMT (Naber et al. 2013). The up-regulation of the transcription factor slug preceded the induction of α-SMA in the UUO model (Lange-Sperandio et al. 2007). Another study suggested that uric acid increased the expression of transcriptional factors, snail1 and slug, thereby contributing to EMT and fibrosis (Ryu et al. 2013). Consistent with these findings, we observed that slug was induced by TGF-β1, which was diminished by the UA treatment. The inhibitory effects of EMT may be partially explained by the decreased expression of snail1 and slug induced by UA.

TGF-β1 mediates several key tubular pathological events, such as EMT, during CKD progression (Lan 2011). TGF-β1 initiates and completes the whole EMT process *in vitro* and *in vivo* under determined conditions (Fan et al. 1999). It is the strongest inducer of EMT in tubule cells. Upon receptor stimulation, activated Smad3 signalling pathways are crucial for EMT through the modulation of the expression of key fibrotic genes (Yang et al. 2010). We found that UA can inhibit the protein expression of TGF-β1 and phosphorylated Smad3, thereby suggesting that UA can inhibit EMT through inhibiting TGF-β1-Smad3 signalling.

## Conclusions

The findings in the present study provide *in vivo* and *in vitro* evidence that UA can attenuate renal tubulointerstitial fibrosis. The anti-fibrotic effects of UA may be achieved by inhibiting Smad3-dependent TGF-β1 signalling, which, in turn, attenuated EMT and fibrogenesis. UA may be an ideal agent for clinical intervention of renal interstitial fibrosis.
